# Machine-Learning–Based Prediction of Biochemical Recurrence in Prostate Cancer Integrating Fatty-Acid Metabolism and Stemness

**DOI:** 10.3390/ijms27020750

**Published:** 2026-01-12

**Authors:** Zao Dai, Ningrui Wang, Mengyao Liu, Zhenguo Wang, Guanyun Wei

**Affiliations:** 1 Research Center for Intelligent Information Technology, Nantong University, Nantong 226019, China; daizao@ntu.edu.cn; 2 Co-Innovation Center of Neuroregeneration, School of Life Sciences, Nantong Laboratory of Development and Diseases, Nantong University, Nantong 226019, China; 3 Department of Statistics and Data Science, The Chinese University of Hong Kong, Shatin, NT, Hong Kong SAR, China

**Keywords:** prostate cancer, machine learning

## Abstract

Prostate cancer (PCa) is a common malignancy among men worldwide. After radical prostatectomy (RP) and radical radiotherapy (RT), patients may experience biochemical recurrence (BCR) of prostate cancer, indicating disease progression. Therefore, it is meaningful to predict and accurately assess the risk of BCR, and a machine-learning-based-model for BCR prediction in PCa based on fatty-acid metabolism and cancer-cell stemness was developed. A stemness prediction model and ssGSEA (single-sample gene set enrichment analysis) empirical cumulative distribution function algorithm were used to score the stemness scoring (mRNAsi) and fatty-acid metabolism of prostate-cancer samples, respectively, and further analysis showed that the two scores of the samples were positively correlated. Based on WGCNA (weighted correlation network analysis), we discovered modules significantly associated with both stemness and fatty-acid metabolism and obtained the genes within them. Then, based on this gene set, 101 algorithm combinations of 10 machine-learning methods were used for training and prediction BCR of PCa, and the model with the best prediction effect was named fat_stemness_BCR. Compared with 23 published PCa BCR models, the fat_stemness_BCR model performs better in TCGA and CPGEA data. To facilitate the use of the model, the trained model was encapsulated into an R package and an online service tool (PCaMLmodel, Version 1.0) was built. The newly developed fat_stemness_SCR model enriches the prognostic research of biochemical recurrence in PCa and provides a new reference for the study of other diseases.

## 1. Introduction

Prostate cancer (PCa) is one of the most common newly diagnosed cancers in men worldwide and the second leading cause of cancer-related death [1], and its incidence and mortality rates are increasing [2,3]. The development of PCa is a multi-stage and evolving process that begins with normal hyperplasia, progresses to prostatic intraepithelial neoplasia, and ultimately evolves into invasive or metastatic cancer. Radical prostatectomy (RP) and radical radiotherapy (RT) are conventional treatment options for most patients with localized PCa. Most primary-prostate-cancer patients can be cured by RP, with a 5-year survival rate of nearly 100%. However, approximately 20–40% of patients who undergo RP and 30–50% of those who receive RT will develop biochemical recurrence (BCR) within 10 years, defined as a serum PSA level > 0.2 ng/mL on at least two consecutive measurements [4]. Biochemical recurrence of PCa means that the patient’s condition has worsened and will develop into castration-resistant prostate cancer (CRPC), which may lead to tumor metastasis and ultimately death.

Studies have shown that biochemical recurrence of prostate cancer is closely related to cellular stemness [5,6]. Cellular stemness refers to the potential of progenitor cells to differentiate and grow into all cell types. The acquisition of stem-cell characteristics and the loss of differentiation are major drivers of tumor progression. Cellular stemness is influenced by various factors, including metabolism. Most tumors rely on glycolysis for energy, while PCa cells are likely to reprogram fatty acid metabolism to obtain energy [7]. Acetyl-CoA carboxylase (ACC), fatty-acid synthase (FASN), ELOVL2 (elongation of very long-chain fatty-acid elongase 2), and SCD (stearoyl-CoA desaturase), which are involved in fatty-acid metabolism, are significantly upregulated in PCa. Moreover, inhibition of FADS1 (fatty-acid desaturase-1) can markedly suppress the proliferation of PCa cells. AR-regulated ELOVL5 is also critical for metastatic PCa, and CRPC exhibits enhanced fatty-acid synthesis and metabolism [8,9,10,11,12,13]. In addition, studies have shown that fatty-acid metabolism is closely associated with cancer-cell stemness, and JAK/STAT-regulated fatty-acid β-oxidation can promote cancer-cell stemness [14,15].

Machine learning has been widely applied in cancer-related research, such as cancer-patient classification, prognosis and survival prediction, tumor gene-regulatory network analysis, tumor evolutionary patterns, tumor subtyping assessment, therapeutic decision-making, and tumor-cell stemness [15,16,17,18]. C. Zhang et al. developed a prostate-cancer stemness-based survival prognostic model using machine-learning approaches such as OCLR (one-class logistic regression) and LASSO (least absolute shrinkage and selection operator) regression [6]. H. Zhao, H. Wang, T. Zhai, and Y. Wang et al. successfully constructed a biochemical recurrence prognostic model based on fatty acid metabolism using LASSO [19,20,21,22]. T. Zhang et al. established a PCa survival prognostic model by integrating stemness-related gene sets with machine-learning algorithms [23].

In this study, we integrated transcriptomic data and biochemical recurrence (BCR) information from 1224 prostate-cancer (PCa) cases obtained from the GEO, CIT, ICGC, TCGA, PCTA, and CPGEA databases. Among these, 786 GEO samples were used as the training set, with 10-fold cross-validation applied during training to perform feature selection and model tuning. The remaining 438 samples from CIT, ICGC, TCGA, PCTA, and CPGEA were used as an independent external testing set for model evaluation. We calculated stemness and fatty-acid-metabolism scores for each PCa sample and found that both scores were elevated in PCa tissues and positively correlated with each other, suggesting that stemness and fatty-acid metabolism may be jointly involved in PCa progression. We constructed a predictive model for PCa BCR by using 101 methods composed of 10 machine-learning algorithms. The optimal model fat_stemness_BCR was compared with other 23 published PCa BCR models, and this model showed better performance. Moreover, the trained model was packaged into an R package (https://github.com/daizao/PCaMLmodel; Version 1.0; accessed on 10 Jan 2026) and deployed as an online prediction tool via Shiny Server (http://www.dzwgylab.com/PCaMLmodel/; Version 1.0; accessed on 10 Jan 2026). The development of this model enriches the research on BCR prediction models for PCa, provides new references for personalized treatment strategies, and offers important clinical insights for patient diagnosis.

## 2. Results

### 2.1. Assessment of Stemness Characteristics in Prostate-Cancer Samples

**Figure 1:**
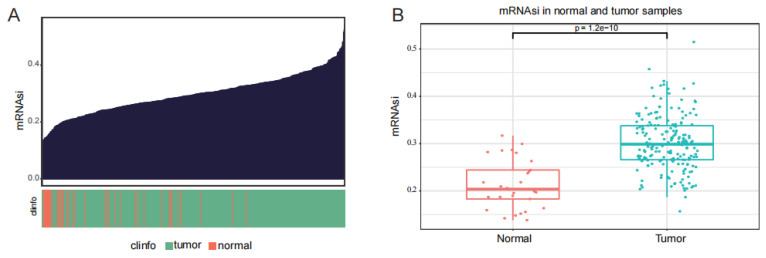
Stemness scores of PCa samples predicted by the stemness model. (**A**) Stemness scores in PCa samples. (**B**) Stemness scores are significantly higher in PCa samples than in normal samples.

A cellular stemness prediction model was established using the OCLR algorithm based on the transcriptome data of pluripotent stem cells and embryonic stem cells from the Progenitor Cell Biology Consortium (PCBC) [24]. The cell stemness prediction model was used to perform stemness scoring (mRNAsi) on the transcriptome data of prostate cancer in the TCGA database (Figure 1A). The results showed that stemness scores in prostate-cancer samples were significantly higher than those in normal prostate samples (Figure 1B).

### 2.2. Assessment of Fatty-Acid Metabolism Characteristics in Prostate Cancer

**Figure 2:**
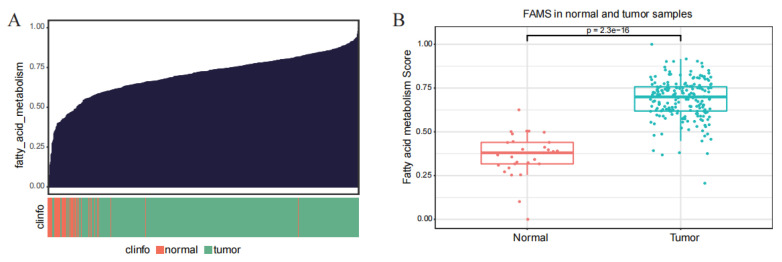
Fatty-acid-metabolism scores of PCa samples. (**A**) Fatty-acid-metabolism scores in PCa samples. (**B**) Fatty-acid-metabolism scores are significantly higher in PCa samples than in normal samples.

Fatty-acid metabolism related genes were obtained from the HALLMARK, KEGG, and REACTOME gene sets, and fatty-acid-metabolism scores (FAMS) for prostate-cancer samples from the TCGA database were calculated using the ssGSEA algorithm (Figure 2A). The fatty-acid-metabolism scores were also significantly higher in prostate-cancer samples (Figure 2B).

### 2.3. Positive Correlation Between Fatty-Acid Metabolism and Stemness Characteristics in Prostate Cancer

**Figure 3:**
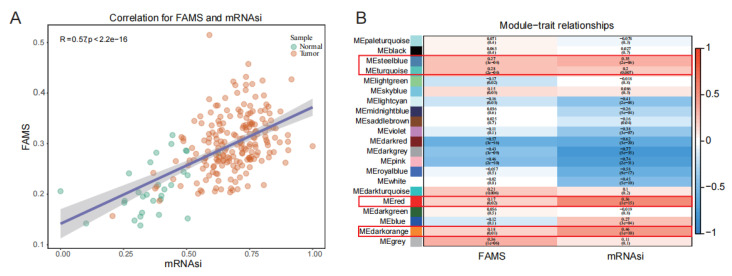
Correlation between stemness and fatty-acid metabolism in prostate cancer and identification of co-expression modules. (**A**) Fatty-acid metabolism is positively correlated with cancer-cell stemness in prostate cancer. (**B**) Co-expression modules associated with fatty-acid metabolism and cancer-cell stemness in prostate cancer.

Pearson correlation analysis between the stemness score and fatty-acid-metabolism score in normal and cancerous samples showed a positive correlation between the two (Figure 3A), indicating that the stemness properties of cancer cells and fatty-acid metabolism can jointly promote the development of prostate cancer. The co-expression similarity algorithm in WGCNA was used to construct a topological overlap matrix (TOM) for module clustering analysis, and four modules (MEsteelblue, MEturquoise, Mered, and MEdarkorange) were identified as being significantly positively correlated with stemness and fatty-acid metabolism (Figure 3B). A total of 1402 genes from these four modules were identified as candidate genes significantly positively associated with fatty-acid metabolism and cancer-cell stemness in prostate cancer.

### 2.4. Construction of a Machine-Learning Prognostic Model for Prostate-Cancer BCR-Integrating Stemness and Fatty-Acid Metabolism

**Figure 4:**
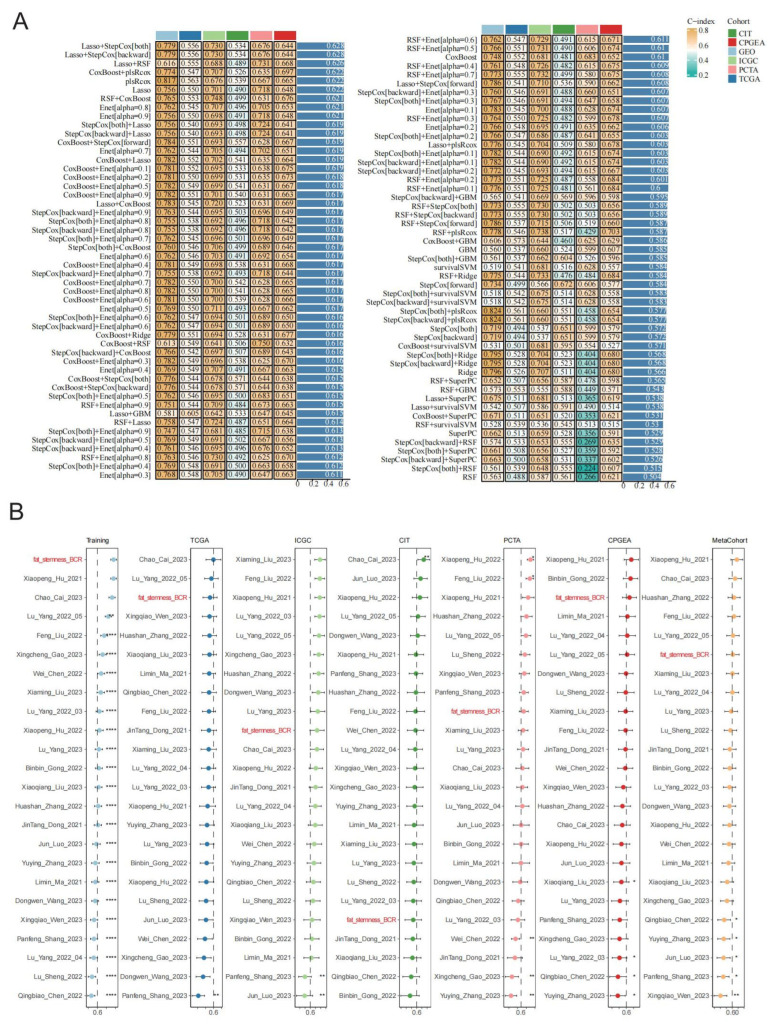
Machine-learning models based on prostate-cancer stemness and fatty-acid metabolism. (**A**) Heatmap of C-index values for 101 combinations of 10 machine-learning algorithms across six datasets (GEO, TCGA, ICGC, CIT, PCTA, and CPGEA). (**B**) Comparison of the optimal model with previously published models based on C-index values across the same datasets. Red indicates the optimal model developed in this study. Each asterisk indicates whether there is a statistically significant difference in the concordance index between our model and other published models (*, 0.01 < *p* ≤ 0.05; **, 0.001 < *p* ≤ 0.01; ****, 0 < *p* ≤ 0.0001).

A total of 1224 prostate-cancer transcriptomic samples with biochemical recurrence (BCR) information were collected from the GEO, CIT, ICGC, TCGA, PCTA, and CPGEA databases. Among these, 786 samples from the GEO database were used as the training set, and 438 samples from the TCGA, ICGC, CIT, PCTA, and CPGEA databases were used as the test set. Five models, Lasso, CoxBoost, RSF, StepCox (both), and StepCox (backward), were employed to perform feature selection for candidate gene sets associated with BCR. In total, 43 publicly available BCR prognostic models for prostate cancer were collected, of which 23 were retained because their required genes were present in the test dataset. Based on the selected genes, 101 model combinations derived from the following 10 machine-learning algorithms were constructed to develop BCR prognostic models for prostate cancer: Lasso, Ridge, Enet (Elastic Net), StepCox (stepwise Cox), survivalSVM (survival support vector machine), CoxBoost, SuperPC (supervised principal component), plsRcox (partial least squares regression for Cox), RSF (random survival forest), and GBM (generalized boosted regression modeling) (Figure 4A). These models were evaluated in the test dataset, and the Lasso + StepCox (both) and Lasso + StepCox (backward) models showed superior performance in prognostic risk scoring. Among all models, the Lasso + StepCox model achieved the best performance for predicting BCR risk in prostate cancer, with a concordance index (C-index) of 0.628; meanwhile, this model also yielded a Brier score of 0.132 (Figure S1). The selected Lasso + StepCox model (with the risk score denoted as fat_stemness_BCR) was compared with 23 previously published BCR prognostic models for prostate cancer using C-index values in the TCGA, ICGC, CIT, PCTA, and CPGEA datasets (Figure 4B). The results showed that our model performed better in the TCGA and CPGEA datasets. In addition, this model incorporates 33 genes: *JAGN1*, *EBPL*, *POLR2H*, *PRPF19*, *CYTH2*, *ZNF532*, *GHDC*, *ZNF696*, *RABGAP1*, *SCAP*, *BCAR1*, *ESRRA*, *PKP3*, *MAEA*, *CFLAR*, *VAMP2*, *MTIF3*, *SHC1*, *CDK7*, *ZCRB1*, *MESP1*, *OSBPL10*, *ATPAF2*, *KPTN*, *DNPEP*, *CHD2*, *AGFG2*, *APEH*, *PSENEN*, *PPTC7*, *LAS1L*, *TRIM35*, and *DOCK1*. Based on data from the Human Protein Atlas (HPA) database [25], 31 of the 33 genes are expressed in cancer cell lines or prostate-cancer tissues (Figure S2). In addition, previous studies have shown that VAMP2 [26] and ZCRB1 [27] are expressed in cancers and promote tumor progression.

To enhance the clinical applicability of the model, we performed multivariable Cox regression analysis by integrating the fat_stemness_BCR risk score with key clinical variables, including prostate-specific antigen (PSA) and Gleason score, to construct a nomogram for predicting BCR (Figure S3A). The total score derived from the nomogram was used to estimate the probability of BCR occurrence at 1 and 3 years. The calibration curves indicated close agreement between the predicted and observed probabilities of BCR at the evaluated time points, demonstrating the predictive accuracy of the model (Figure S3B,C). Decision curve analysis showed that the fat_stemness_BCR score yielded a higher net benefit for predicting BCR compared with PSA and Gleason score (Figure S3D,E). The AUC curves showed that the model achieved higher predictive performance for BCR than PSA and Gleason score, suggesting its potential clinical applicability (Figure S3F).

### 2.5. Development of an R Package and Online Tool for BCR Prediction in Prostate Cancer

**Figure 5:**
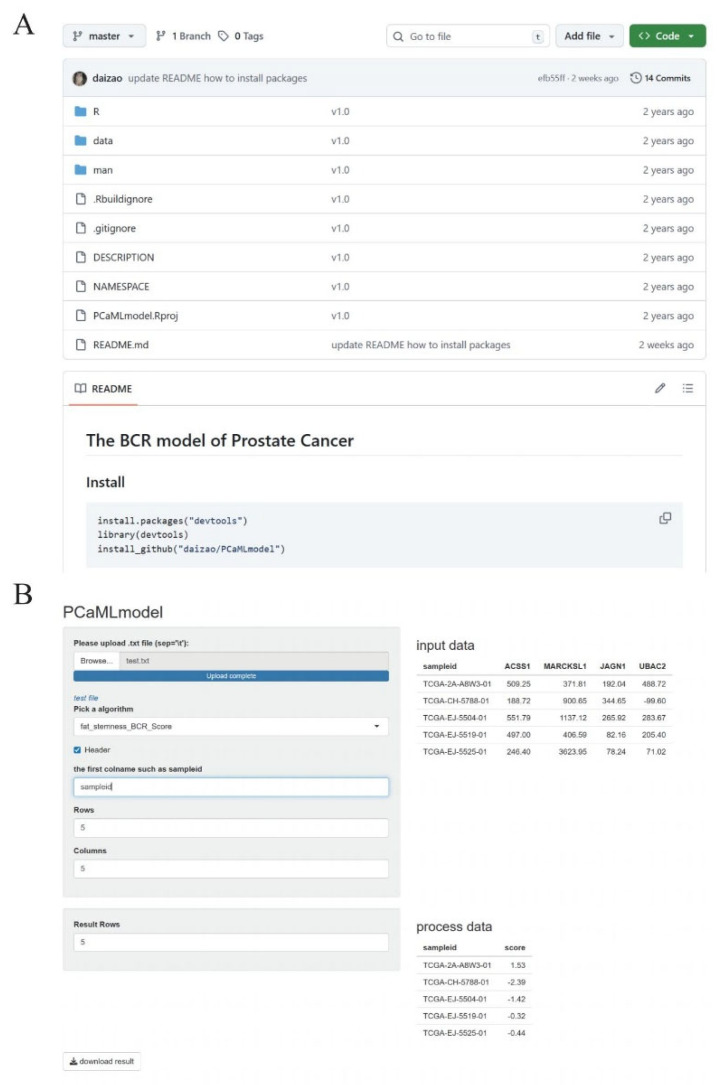
The R package and online prediction tool are based on the prostate-cancer BCR prognostic model. (**A**) The R package for the model is stored on GitHub. (**B**) The web-based online service for the model (providing prognostic analysis) is accessible online.

To facilitate the application of the prostate-cancer BCR prognostic model, the trained model was packaged into an R package (https://github.com/daizao/PCaMLmodel; Version 1.0; accessed on 10 Jan 2026) (Figure 5A) and deployed as an online web service (http://www.dzwgylab.com/PCaMLmodel/; Version 1.0; accessed on 10 Jan 2026) (Figure 5B).

## 3. Discussion

Radical prostatectomy (RP) and radiotherapy (RT) are the standard treatment options for most patients with prostate cancer (PCa). However, patients who receive these treatments may still experience biochemical recurrence (BCR), indicating disease progression. Postoperative BCR is a critical turning point for determining whether to adjust the treatment strategy for patients with primary PCa; therefore, identifying patients at high risk of BCR is essential. Clinicians often combine multiple clinical variables to construct prognostic models, but the predictive accuracy of these models remains limited. To address this issue, we developed a machine-learning-based predictive model using a large-scale prostate-cancer dataset to identify patients at high risk of BCR.

Cell stemness refers to the capacity of cells to proliferate and differentiate into various cell types. The acquisition of stemness and the loss of differentiation characteristics are major drivers of tumor progression. Cell stemness is influenced by multiple factors, including metabolism. While most tumors rely on glycolysis for energy, prostate-cancer (PCa) cells tend to obtain energy through reprogrammed fatty-acid metabolism [28]. Furthermore, castration-resistant prostate cancer (CRPC) also exhibits enhanced fatty-acid synthesis and metabolism [11]. Therefore, it is feasible to construct a predictive model for patients at high risk of biochemical recurrence (BCR) by combining features related to PCa cell stemness and fatty-acid metabolism with machine-learning algorithms.

In this study, a total of 1224 PCa transcriptome samples with BCR clinical information were collected from multiple public cancer databases, including GEO, CIT, ICGC, TCGA, PCTA, and CPGEA. During model development, strict separation between the training and testing datasets was maintained. In addition, validation across multiple independent cohorts may help reduce the risk of model overfitting and cohort-specific bias. Among the 101 algorithm combinations evaluated, the combination of Lasso and StepCox was ultimately identified as the optimal approach for predicting BCR risk in PCa, achieving a C-index of 0.628. Compared with conventional clinical indicators, our model showed favorable predictive performance. In contrast to earlier studies that were often based on single-cohort analyses with limited validation, our approach leveraged multi-cohort integration and systematic validation, which may improve the robustness of the findings. The resulting model uses 33 marker genes, which show almost no overlap with the marker genes included in the three PCa prognostic models recommended by the National Comprehensive Cancer Network guidelines, except for RABGAP1 [29,30,31]. Compared with other published models, our model demonstrates superior predictive performance in the TCGA and CPGEA datasets. To facilitate clinical application, the trained model has been implemented as an R package, and an online prediction tool has been deployed, providing an easy-to-use platform for BCR risk assessment in PCa. In future work, we plan to incorporate additional machine-learning and deep-learning methods, expand the PCa cohorts, and further improve the prediction accuracy and clinical applicability of the model.

This study highlights the roles of cancer-cell stemness and fatty-acid metabolism in the progression of prostate cancer and provides a machine-learning-based tool for prognostic risk stratification in PCa. Furthermore, it suggests potential molecular targets for future therapeutic intervention. Nevertheless, several limitations should be acknowledged. Although this study integrated multiple public datasets and employed various machine-learning algorithms for model construction, functional experiments and prospective clinical validation are still lacking, which limits direct confirmation of the clinical applicability of the proposed model.

## 4. Materials and Methods

### 4.1. Study Design

**Figure 6:**
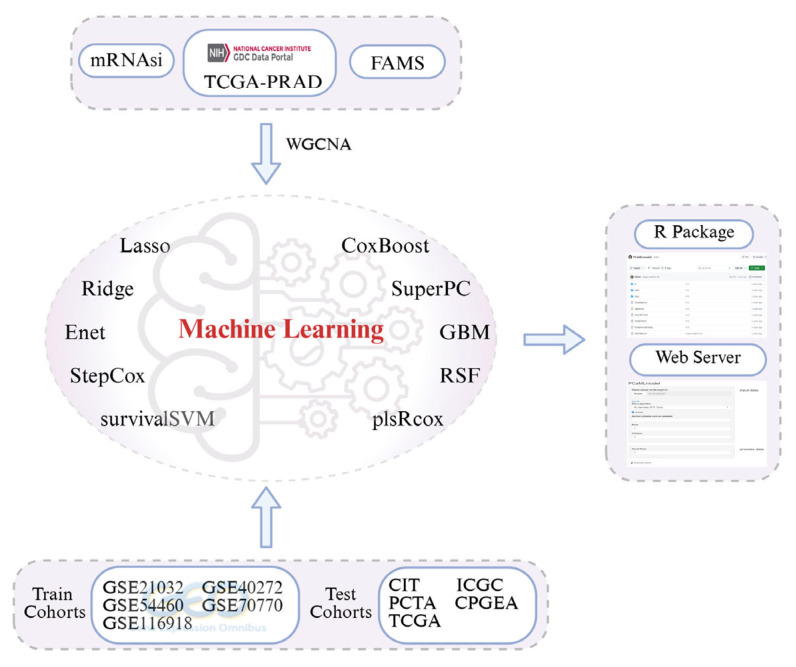
Overall workflow of the study. In this work, ten machine-learning algorithms were applied to train and test predictive models using data from 1224 prostate-cancer patients. The final models were subsequently implemented as an R package and deployed as an online web-based service to facilitate broader application.

The overall workflow is illustrated in Figure 6. Prostate-cancer samples were first collected from multiple databases, and stemness scores and fatty-acid-metabolism scores were calculated for each sample. Based on these data, we identified a gene set associated with BCR prognosis in prostate-cancer and constructed-prognostic models using multiple machine-learning algorithms. Finally, the best-performing model was implemented as an R package and deployed as an online prediction tool.

### 4.2. Data Collection and Processing

The transcriptome data of 1224 PCa cases with biochemical recurrence (BCR) information were obtained from the GEO, CIT, ICGC, TCGA, PCTA, and CPGEA databases. Transcriptome data from 786 PCa cases with BCR information in GEO were used as the training set, and data from 438 PCa cases with BCR information in the TCGA, ICGC, CIT, PCTA, and CPGEA cohorts were used as the external test set. Among these datasets, GSE21032 and CIT were obtained from raw microarray data; GSE40272, GSE70770, and GSE116918 were obtained from SOFT-formatted data; and GSE54460, TCGA, ICGC, PCTA, and CPGEA were obtained from processed transcriptomic matrix files.

Data were included and processed according to the following criteria and steps: (1) tissue samples were derived from patients with PCa; (2) clinical information on biochemical recurrence was available; (3) for GEO, expression profile data of PCa were obtained, and multiple GEO datasets were merged and adjusted for batch effects; (4) for CIT, raw microarray data were normalized and expression values were transformed. For the CIT, ICGC, TCGA, PCTA, and CPGEA datasets, expression data were then processed as follows: the mean expression level was calculated for genes with multiple probes or duplicate gene names, and RNA-seq data (ICGC, TCGA, and CPGEA) were converted from FPKM or raw counts to TPM matrices. Batch effects across all datasets were corrected using the ComBat function in the sva R package with default parameters [32].

### 4.3. Cell Stemness Prediction Model

Based on the transcriptomic data of pluripotent stem cells and embryonic stem cells from the Progenitor Cell Biology Consortium (PCBC) [24], the stemness score was calculated using a model constructed based on the OCLR (one-class logistic regression) algorithm, as previously described [33]. OCLR is an extension of conventional logistic regression, with its core principle being the construction of a model by maximizing the similarity among target samples while incorporating regularization to prevent overfitting. The optimization objective of this algorithm is to maximize the average log-likelihood with a regularization term, which can be formulated as follows:

(1) $$\max_{\omega }\frac{1}{n}\sum_{i=1}^{n}\left[\omega^{T}x_{i}-\log\left(1+e^{\omega^{T}x_{i}}\right)\right]-\lambda R(\omega )x$$

In this formulation, $x_{i}$ represents the d-imensional gene expression vector, ω denotes the d-dimensional weight parameter, λ is the regularization parameter, and $R\left(\omega \right)$ is the regularization function, with L2 regularization applied by default. As the objective function is a continuously differentiable convex function, it is optimized iteratively using the Newton–Raphson method. Through a Taylor expansion, the nonlinear optimization problem is transformed into a weighted linear regression, and the weight parameter ω is updated iteratively until convergence of the objective function is achieved.

### 4.4. Fatty-Acid Metabolism Scoring

The fatty-acid metabolism-related genes were obtained from the HALLMARK, KEGG, and REACTOME databases, and the corresponding fatty-acid-metabolism score was calculated based on the ssGSEA (single-sample gene set enrichment analysis) [34] algorithm using these genes. The core principle of ssGSEA is to quantify the absolute enrichment level of a specific gene set within an individual sample, thereby reflecting the activation status of the gene set in that sample. The enrichment score (ES) is derived by integrating the difference between two empirical cumulative distribution functions (ECDFs), which quantify whether genes in the target gene set G are preferentially distributed toward the top of the ranked gene list L. The two key ECDFs are defined as follows: (1) the weighted cumulative distribution function of the target gene set, $P_{G}^{\omega }$:

(2) $$P_{G}^{\omega }\left(G,S,i\right)=\sum_{r_{j}\in G,j\leq i}\frac{{\left|r_{j}\right|}^{2}}{\sum_{r_{j}\in G}{\left|r_{j}\right|}^{\alpha }}$$

In this definition, the numerator represents the sum of the squared ranks of the top *i* genes within the target gene set G, while the denominator corresponds to the sum of the squared ranks of all genes in G. The parameter α is set to 1/4 by default and is used to adjust the weighting strength, thereby balancing the contributions of high-ranked and low-ranked genes. (2) The cumulative distribution function of the non-target gene set, denoted as $P_{N_{G}}$, is defined as follows:

(3) $$P_{N_{G}}(G,S,i)=\sum_{r_{j}\notin G,j⩽i}\frac{1}{N-N_{G}}$$

In this formulation, the numerator represents the number of non-target genes appearing among the top *i* genes in the ranked list, while the denominator denotes the total number of genes in the non-target gene set. The enrichment score (ES) is then calculated as the cumulative difference between $P_{G}^{\omega }$ and $P_{N_{G}}$, which is defined as follows:

(4) $$ES\left(G,S\right)=\sum_{i=1}^{N}\left[P_{G}^{\omega }\left(G,S,i\right)-P_{N_{G}}\left(G,S,i\right)\right]$$

A larger value $ES\left(G,S\right)$ indicates a higher degree of enrichment.

### 4.5. WGCNA

Co-expressed gene modules linked to fatty-acid metabolism and stemness in prostate cancer were identified via weighted correlation network analysis (WGCNA), together with a co-expression similarity algorithm and hierarchical clustering methods.

### 4.6. Construction Prostate-Cancer BCR Prognostic Model Using Machine Learning

Transcriptomic data from PCa cases with BCR clinical information in the GEO database were used as the training set, whereas data from the CIT, ICGC, TCGA, PCTA, and CPGEA databases were used as the test set. A BCR prognostic model for prostate cancer was developed using 10 different combinations of machine-learning algorithms (e.g., Lasso + StepCox), including Lasso, Ridge, Enet (elastic net), StepCox (stepwise Cox), survivalSVM (survival support vector machine), CoxBoost (Cox model by likelihood-based boosting), SuperPC (supervised principal components), plsRcox (partial least squares regression for Cox), RSF (random survival forest), and GBM (generalized boosted regression modeling). The R packages used for the machine-learning algorithms in this study included survival, randomForestSRC, glmnet, plsRcox, superpc, gbm, mixOmics, survcomp, CoxBoost, survivalsvm, and BART. The analytical workflow of this study was informed by a publicly available online resource (FigureYa) [35], which provides a conceptual overview of the applied methods. During model training, 10-fold cross-validation was employed to perform feature selection and model tuning. Parameter optimization involved obtaining the optimal λ values for Lasso, Ridge, and Elastic Net (Enet); the optimal penalty parameter for CoxBoost; the optimal threshold for SuperPC; the optimal nt parameter for plsRcox; and the optimal n.trees for GBM. In addition, a grid search approach was applied to select the best direction and α parameters for the StepCox and Enet models, respectively, ensuring optimal model performance. In each fold, the data were automatically partitioned into training and validation subsets, and this procedure was conducted exclusively within the training set. The testing set was not involved in any step of model training or optimization. Model performance was further evaluated using time-dependent Brier scores to quantify the accuracy of probabilistic predictions at 12, 24, and 36 months in the independent testing cohorts. After completion of the training process, feature genes associated with the prediction of biochemical recurrence (BCR) in prostate cancer were extracted from the optimal model according to the features retained in the final optimized model. Representative R code examples illustrating the key analytical steps are provided in the Supplementary Materials Code S1.

### 4.7. Nomogram Construction

A nomogram model integrating PSA (prostate-specific antigen), Gleason score, and the fat_stemness_BCR score was constructed to predict biochemical recurrence (BCR) in prostate cancer. Calibration curves, decision curve analysis, and ROC curves were used to evaluate the predictive accuracy of the model.

### 4.8. R Package and Online Tool

The PCa BCR prognostic model was packaged into an R package (https://github.com/daizao/PCaMLmodel; Version 1.0; accessed on 10 Jan 2026) and deployed an online service using Shiny Server (http://www.dzwgylab.com/PCaMLmodel; Version 1.0; accessed on 10 Jan 2026).

### 4.9. Statistical Analysis

All statistical analyses and data visualizations were performed using R. Group comparisons were conducted using two-sample *t*-tests, and correlations were assessed using Pearson’s product–moment correlation coefficient. A *p* value < 0.05 was considered statistically significant.

## 5. Conclusions

This study demonstrates a close relationship between cancer-cell stemness and fatty-acid metabolism, both of which contribute to the progression of prostate cancer (PCa) and can be leveraged for prognostic assessment. Using a large-scale transcriptomic dataset and machine-learning algorithms, we constructed a prognostic model for biochemical recurrence (BCR) in PCa. The Lasso–StepCox model was systematically evaluated using independent external cohorts and demonstrated reasonable predictive performance in a clinical context. Furthermore, the model has been successfully implemented as an R analysis package and an online prediction tool. Our work provides new references and practical tools for clinical BCR risk assessment and individualized prognostic evaluation in PCa.

## Data Availability

The original contributions presented in this study are included in the article/Supplementary Materials. Further inquiries can be directed to the corresponding author.

## Supplementary Materials

The supporting information can be downloaded at: https://www.mdpi.com/article/10.3390/ijms27020750/s1.

## Abbreviations

PCa	prostate cancer
RP	radical prostatectomy
RT	radiotherapy
BCR	biochemical recurrence
mRNAsi	stemness scoring
PSA	serum prostate-specific antigen
CRPC	castration-resistant prostate cancer
OCLR	one-class logistic regression
LASSO	least absolute shrinkage and selection operator
PCBC	Progenitor Cell Biology Consortium
WGCNA	weighted correlation network analysis
ssGSEA	single-sample gene set enrichment analysis
FAMS	fatty-acid-metabolism score
