# Supplementary material for: Machine-Learning–Based Prediction of Biochemical Recurrence in Prostate Cancer Integrating Fatty-Acid Metabolism and Stemness
Source: Int J Mol Sci. 2026 Jan 12;27(2):750. doi: 10.3390/ijms27020750 (PMC12841470; doi:10.3390/ijms27020750)
Supplement: Supplementary file 1 [file ijms-27-00750-s001.zip › Code S1.pdf]

```r
## Example 1: Data preprocessing and batch correction

library(sva)
library(limma)

# Expression matrix (genes × samples)
expr <- as.matrix(expr_data)

# Batch information and clinical covariates (include bcr_time,bcr_fustat)
batch <- total_clin$batch
design <- model.matrix( ~ BCR_fustat,data=total_clin)

# Batch-effect correction using ComBat
expr_combat <- ComBat(dat = expr,
                      batch = batch,
                      mod = design)

# scale data if needed
input_data <- scale(t(expr_combat))


## Example 2: Lasso-StepCox model training

library(glmnet)
library(survival)

# Survival object
y <- Surv(time = train_clin$BCR_time, event = train_clin$BCR_fustat)

# Lasso Cox regression with 10-fold cross-validation
lasso_cv.fit <- cv.glmnet(x = input_data,
                          y = y,
                          family = "cox",
                          alpha = alpha,
                          nfolds = 10)
lasso.fit <- glmnet(x = input_data,
                    y = y,
                    family = "cox",
                    alpha = alpha,
                    lambda = lasso_cv.fit$lambda.min)

# Extract features (lasso)
lasso_coef <- coef(lasso.fit)
selected_genes <- rownames(lasso_coef)[which(lasso_coef[,1] != 0)]

# Stepwise Cox regression
cox_data <- data.frame(input_data[, selected_genes],
                       time = train_clin$BCR_time,
                       status = train_clin$BCR_fustat)

stepcox.fit <- step(coxph(formula = Surv(time, status) ~ ., data = cox_data),
                    direction = "both", trace = 0)
```

```r
# Extract final features
features_genes <- names(coef(stepcox.fit))

## Example 3: Risk score calculation in an external cohort

# External expression matrix (genes × samples)
expr_test <- t(as.matrix(expr_test_data))

# Calculate risk score
risk_score <- predict(stepcox.fit, type = 'lp', as.data.frame(expr_test)),


## Example 4: Model performance evaluation

library(survcomp)
library(pec)

# C-index
test_clin$score <- as.vector(risk_score)
c_index <- summary(coxph(formula = Surv(BCR_time,BCR_fustat) ~ score,
                         data = test_clin))$concordance["C"]

# Brier score at predefined time points
brier <- pec(object = stepcox.fit,
             formula = Surv(BCR_time, BCR_fustat) ~ score,
             data = test_clin,
             times = c(12, 24, 36))
```
