# Supplementary material for: Machine-Learning–Based Prediction of Biochemical Recurrence in Prostate Cancer Integrating Fatty-Acid Metabolism and Stemness
Source: Int J Mol Sci. 2026 Jan 12;27(2):750. doi: 10.3390/ijms27020750 (PMC12841470; doi:10.3390/ijms27020750)
Supplement: Supplementary file 1 [file ijms-27-00750-s001.zip › Figure S1-S3.pdf]

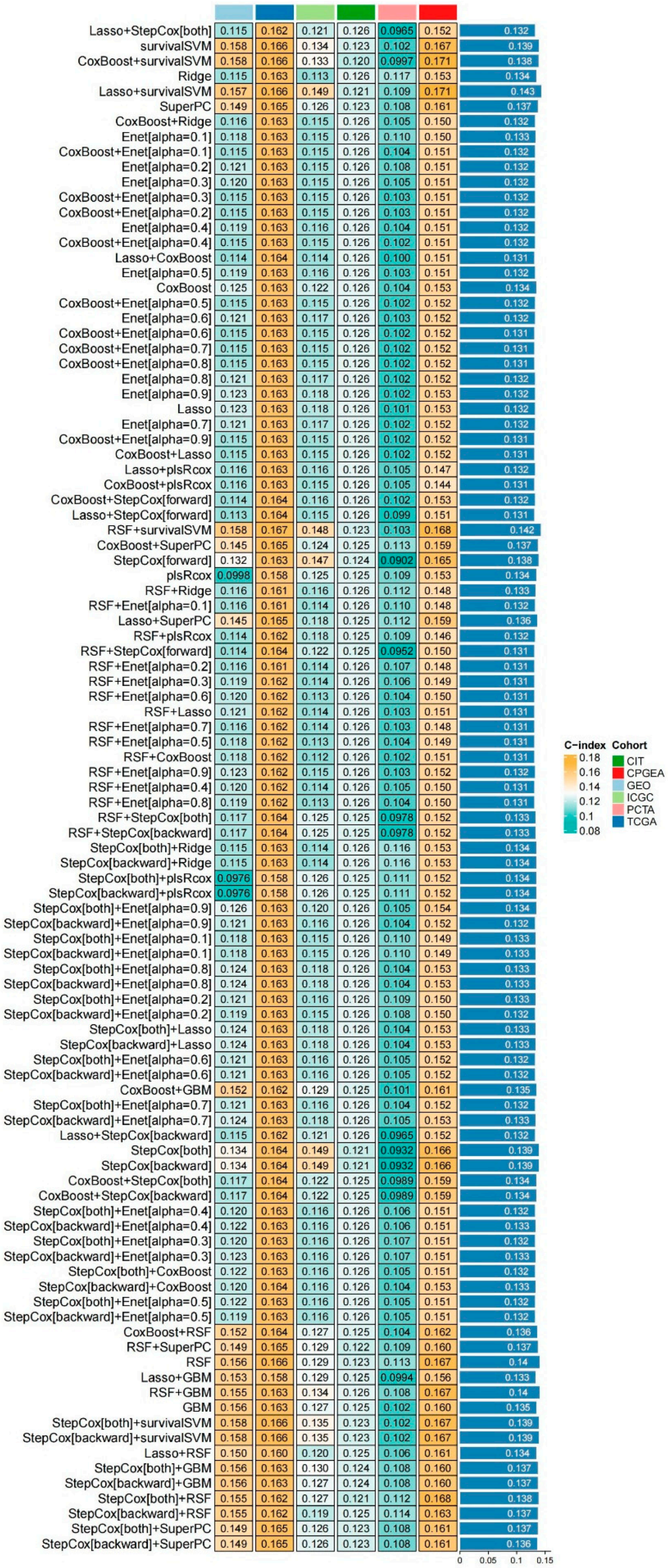

Figure S1. Heatmap of Brier Score for 101 combinations of 10 machine-learning algorithms across six datasets (GEO, TCGA, ICGC, CIT, PCTA, and CPGEA).

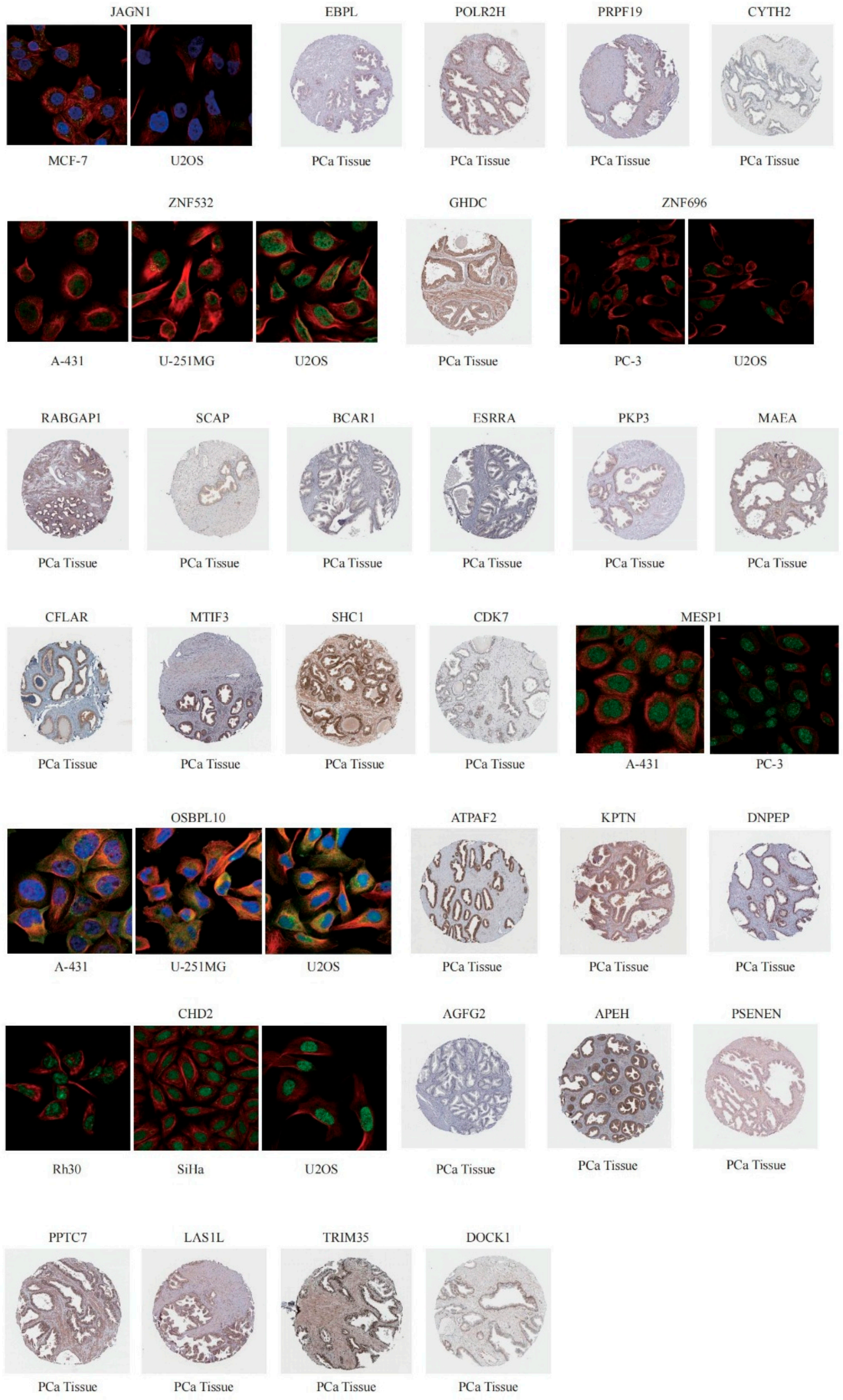

Figure S2. Expression of 31 genes in cancer-cell lines and prostate-cancer tissues based on the Human Protein Atlas (HPA) database.

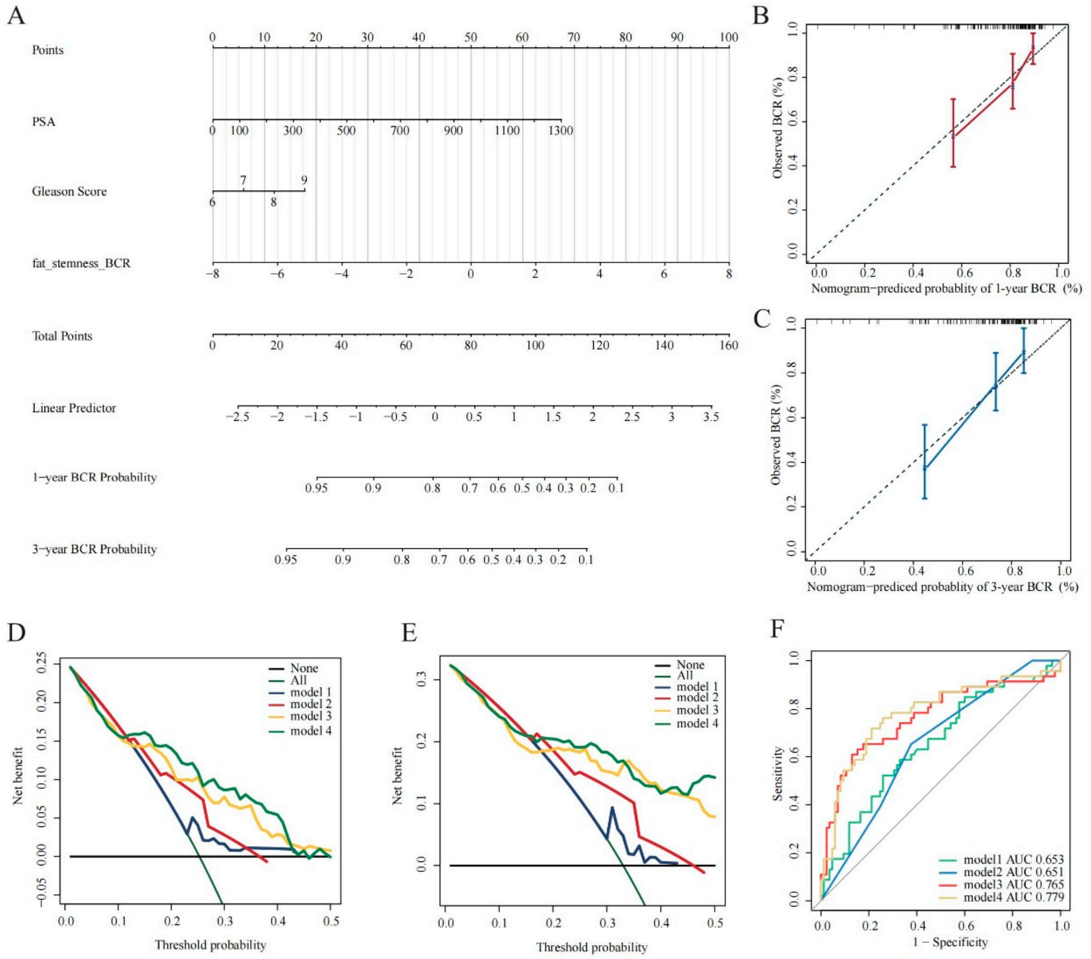

Figure S3. Prediction of biochemical recurrence (BCR) risk based on clinical characteristics and the fat_stemness_BCR model score. (A) A nomogram constructed using a multivariable Cox proportional hazards regression model integrating the fat_stemness_BCR score, Gleason score, and prostate-specific antigen (PSA) for BCR risk prediction. (B, C) Calibration curves for predicting 1-year and 3-year BCR probability. (D, E) Decision-curve analysis for 1-year and 3-year BCR prediction. (F) Receiver operating characteristic (ROC) curves comparing the predictive performance of different models. model 1: PSA-based prediction model; model 2: Gleason score-based prediction model; model 3: fat_stemness_BCR-based prediction model; model 4: integrated prediction model combining PSA, Gleason score, and fat_stemness_BCR; AUC: Area Under Curve.
